# Synthesis of Fluorescent Dansyl Derivatives of Methoxyamine and Diphenylhydrazine as Free Radical Precursors

**DOI:** 10.3390/ijms21103559

**Published:** 2020-05-18

**Authors:** Bianca Patrascu, Sorin Mocanu, Anca Coman, Augustin M. Madalan, Codruta Popescu, Anca Paun, Mihaela Matache, Petre Ionita

**Affiliations:** 1 Department of Organic Chemistry, Biochemistry and Catalysis, Faculty of Chemistry, University of Bucharest, Panduri 90-92, 050663 Bucharest, Romania; biancapatrascu92@yahoo.ro (B.P.); coman_anca2006@yahoo.com (A.C.); c.paraschivescu@gmail.com (C.P.); ancaarnautu@yahoo.com (A.P.); mihaela.matache@g.unibuc.ro (M.M.); 2 Institute of Physical Chemistry, Spl. Independentei 202, 060021 Bucharest, Romania; sorin.mocanu89@gmail.com; 3 Department of Inorganic Chemistry, Faculty of Chemistry, University of Bucharest, Dumbrava Rosie 23, 020462 Bucharest, Romania; augustin.madalan@chimie.unibuc.ro

**Keywords:** dansyl, fluorescence, free radical, hydrazyl

## Abstract

Starting from dansyl-chloride, in reaction with 1,1-diphenylhydrazine and methoxyamine, two new fluorescent derivatives **1** and **2** were obtained and characterized by NMR, IR, UV-Vis, HR-MS, and fluorescence spectroscopy. The single-crystal X-ray structure was obtained for compound **2**. Both compounds generate free radicals by oxidation, as demonstrated by ESR spectroscopy. Compound **1** generates the corresponding hydrazyl-persistent free radical, evidenced directly by ESR spectroscopy, while compound **2** generates in the first instance the methoxyaminyl short-lived free radical, which decomposes rapidly with the formation of the methoxy radical, evidenced by the ESR spin-trapping technique. By oxidation of compounds **1** and **2**, their fluorescence is quenched.

## 1. Introduction

**Figure 1:**
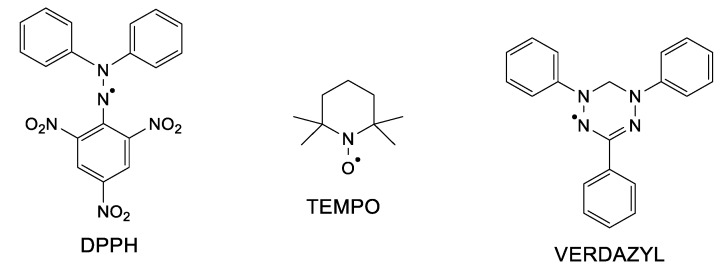
Chemical structure of a hydrazyl, nitroxide, and a verdazyl free radical.

Hydrazyl free radicals are a class of organic compounds that possess astonishing properties. First of all, the unpaired electron (free electron) makes such compounds paramagnetic; secondly, the open-shell structure brings high reactivity [1]. For example, hydrazyl radicals easily participate in redox reactions (electron transfer) or in acid–base ones (proton transfer). Besides, such reactions are paired with color changes, making it very easy to follow even by the naked eye. Many of the hydrazyl free radicals are persistent or stable under usual conditions. 2,2,-Diphenyl-1-picrylhydrazyl free radical, well known as DPPH (Figure 1), is a solid soluble in organic solvents, developing a purple color. Due to its stability and to the fact that, either in solid state or in solution, it is a 100% free radical (does not dimerize nor react with oxygen), it is used as a standard in electron spin resonance spectroscopy (ESR or EPR) [2]. DPPH was firstly synthesized and assumed as a free radical in 1922 [3], at a time when ESR or free radical chemistry had not been developed yet. Decades later, hydrazyl chemistry gained great development. Although these free radicals hold important properties, they seem to almost be forgotten nowadays among the nitroxides and verdazyls classes of stable free radicals (see also Figure 1), which are much often encountered in the literature [4,5].

Methoxyaminyl free radicals are another class of persistent free radicals stabilized by the push–pull or capto-dative effect [6,7]. These are important intermediates in the synthesis of highly colored compounds of diazenium betaine type [8].

Dansyl-chloride (5-(dimethylamino)naphthalene-1-sulfonyl chloride) is a highly reactive non-fluorescent reagent that easily reacts with amines, yielding fluorescent derivatives; the reaction is widely used for amino-acids derivatization and many other detection techniques [9,10,11,12].

The free electron present in open-shell molecules, which are free radicals, is a strong quencher for fluorescence. Based on this behavior, a novel class of hybrid organic compounds, containing a free radical and a fluorescent moiety in the same molecule, has been developed under the usual name of pro-fluorescent free radicals. As fluorescent moieties, 5-(dimethylamino)naphthalene-1-sulfonyl (dansyl), pyrene, or 7-nitrobenzofurazan (NBD) motifs are usually used. Pro-fluorescent free radicals are in the first-instance paramagnetic compounds, which show no or weak fluorescence (due to the unpaired electron, often named spin), but after one electron reduction, they become highly fluorescent. Nitroxides or verdazyl free radicals were frequently used for this purpose [13,14,15].

**Figure 2:**
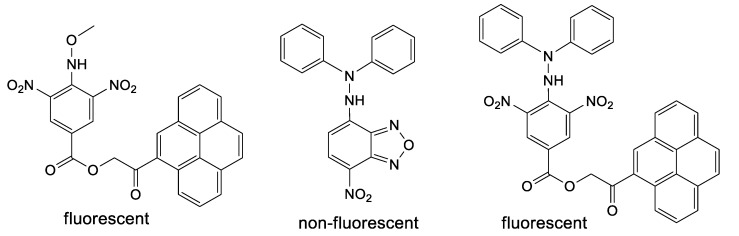
Methoxyamine and hydrazine derivatives, precursors of persistent free radicals, containing a pyrene or NBD fluorophore.

Pro-fluorescent hydrazyls are not present or studied in the literature to the best of our knowledge. However, few precursors of such type (Figure 2) can be found [16,17]; interestingly, for such compounds, only pyrene derivatives are fluorescent, while the nitrobenzofurazan derivative is not fluorescent [18]. Similarly, aromatic sulfonyl diphenylhydrazines as precursors of hydrazyl free radicals are very rarely encountered in the literature [19,20,21], while methoxysulfonoamides as precursors of methoxyaminyl free radicals are not at all.

## 2. Results and Discussion

### 2.1. Synthesis of Dansyl Derivatives 1 and 2 and Structural Characterization

**Figure 3:**
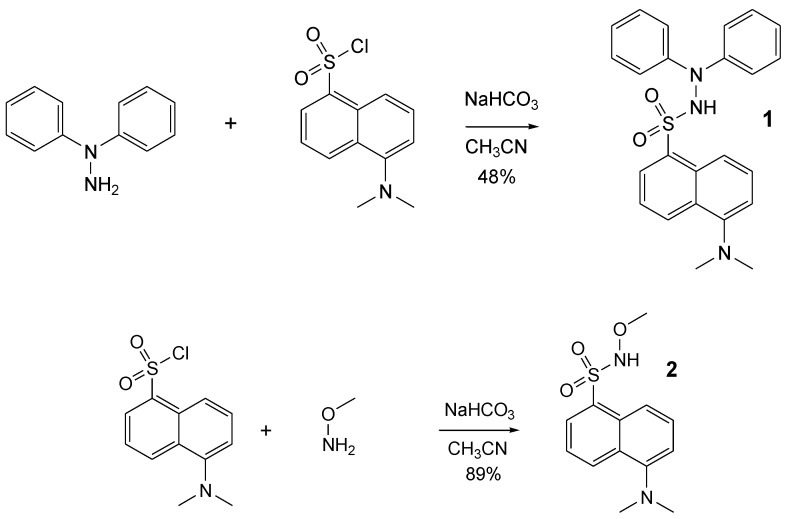
Synthesis of the compounds **1** and **2.**

Following our interest and experience in methoxyaminyl [22] and hydrazyl free radicals [18], we aimed to synthesize two new compounds, derived from dansyl chloride, presuming that they can be easily converted into pro-fluorescent persistent free radicals. Thus, dansyl chloride in reaction with 1,1-diphenylhydrazine or methoxyamine afforded the expected compounds **1** and **2** (Figure 3). Reactions were performed at room temperature, in a single step, with 48% and 89% yields, respectively.

The IR spectra of compounds **1** and **2** showed the expected bands of amino groups between 3400 and 3100 cm^−1^, aromatic CH at 3100–3000 cm^−1^, methyl groups at 3000–2900 cm^−1^, and nitro groups around 1550 and 1350 cm^−1^, while the SO_2_ group yields bands with maxima at about 1150 cm^−1^.

The structure of these compounds was also confirmed by ^1^H- and ^13^C-NMR. The N*H* signal can be found at 7.73 ppm for **1** and 7.38 ppm for **2** (in CDCl_3_); methyl groups at 2.80 and 2.93 ppm, for **1** and **2**, respectively; and for **2**, the methoxy group is found at 3.69 ppm.

HR-MS of both compounds **1** and **2** also confirmed their structure.

**Figure 4:**
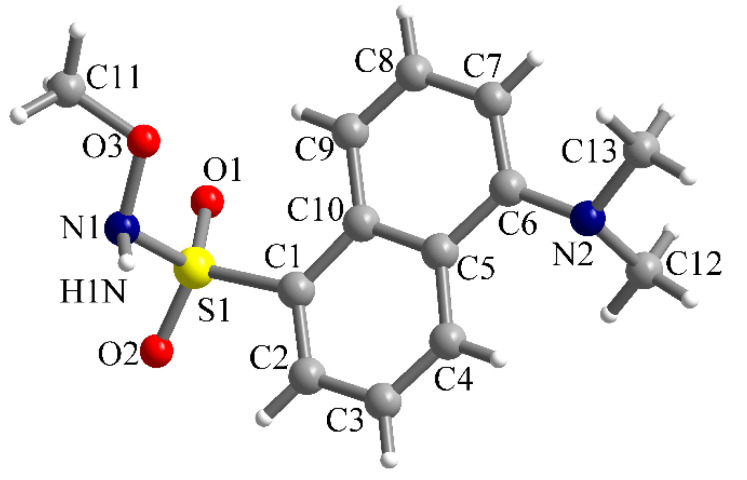
View of the molecular structure of compound **2** along with atoms labelling scheme.

In the case of the methoxy-derivative **2**, crystals suitable for X-ray diffraction were obtained. Compound **2** crystallizes in the monoclinic *P2*_1_/*n* space group and the molecular structure resulting from X-ray diffraction on a single crystal is presented in Figure 4. In the sulfonyl group, the S=O bond lengths are S1-O1 = 1.4201(14) and S1-O2 = 1.4229(14) Å. The S1-C1 and S1-N1 bond lengths are 1.7842(16) and 1.6601(16) Å, respectively. Other bond lengths are gathered in Table 1.

**Figure 5:**
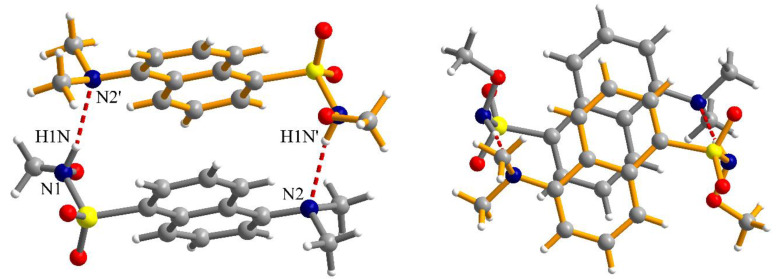
Two different views of the supramolecular dimers formed by hydrogen bonding and π–π interactions in crystal **2** (symmetry code: ’ = 1–x, 2–y, –z); views are perpendicular to each other.

**Figure 6:**
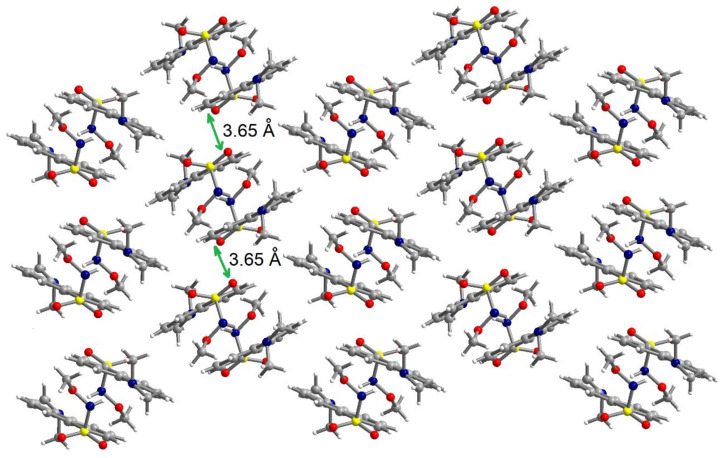
View of a packing diagram in crystal **2**.

The N-H fragment of the methoxysulfonoamide group is involved as a donor in hydrogen interaction with the amino group of a neighboring molecule generating supramolecular dimers, N1-H1N···N2′ = 2.13 Å, symmetry code: ’ = 1–x, 2–y, –z (Figure 5). The formation of supramolecular dimers is also reinforced by π–π interactions established between the naphthalene fragments. The separation between the aromatic fragments within the supramolecular dimers is 3.52–3.55 Å. Analysis of the packing diagrams suggested weaker π–π interactions that could be observed between the supramolecular dimers of the aromatic fragments and separation of 3.65 Å (Figure 6).

### 2.2. Photo-Physical Properties

**Figure 7:**
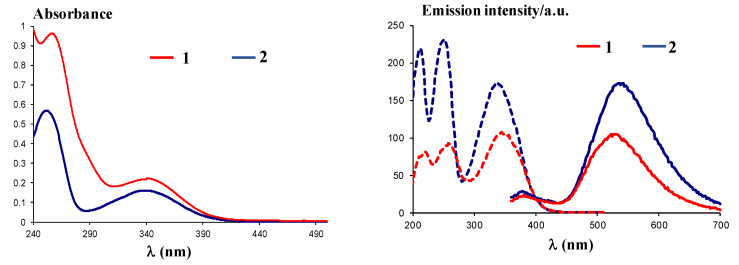
Left: UV-Vis spectra of compounds **1** and **2** in ethanol; right: Excitation (dotted lines) and emission (plain lines) spectra of compounds **1** and **2**.

UV-Vis spectra of compounds 1 and 2 recorded in ethanol at a concentration of 5 × 10^−5^ M showed similar absorption maxima and have the following characteristics: Compound 1 (Figure 7, left) has two bands with absorption maxima at λ_max_ = 262 nm and λ_max_ = 346 nm, while compound **2** (Figure 7, left) has the absorption maxima at λ_max_ = 254 nm and λ_max_ = 340 nm, confirming the absorption behavior of the dansyl moiety [23].

Excitation at λ_ex_ = 340 nm of the solutions in ethanol for compounds **1** and **2** 10^−4^ M led to a maximum emission of λ_em_ = 530 nm for compound **1** and λ_em_ = 535 nm for compound **2**, similar to related compounds [24]. The fluorescence behavior could be assigned to the dansyl moiety, known for the typical charged-transfer band between the donor dimethylamino group and the sulfonyl unit, centered around λ_em_ = 540 nm, as previously described [23]. In addition, use of polar solvents affords better emission, as found before [23], and justified the use of ethanol to yield high green emission intensities, with Stokes shifts of 184 nm (10.03 × 10^−4^ cm^−1^) and 195 (10.72 × 10^−4^ cm^−1^), respectively.

### 2.3. Formation of Free Radicals

**Figure 8:**
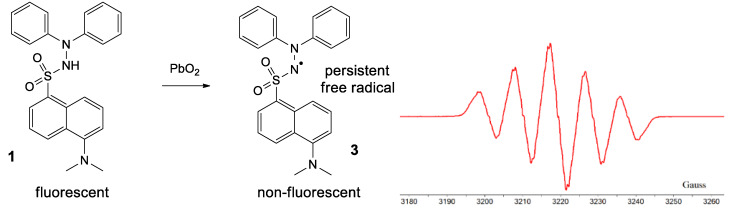
Formation of **3** and its ESR spectrum.

By treating 1 with lead dioxide, it was oxidized to the corresponding persistent free radical **3** (Figure 8). This was characterized by ESR. The general feature of the spectrum consists of five lines with relative intensities of 1/2/3/2/1, with hyperfine coupling constants of *a_N1_ = a_N2_* = 9.25 Gauss (G) and very broad lines (linewidth 4 G), which is typical for most hydrazyls (a reported compound [19], similar to 3, *N’,N’*-diphenyl-benzenesulfono-hydrazyl free radical, has *a_N1_ = a_N2_* = 9.4 G). In time, 3 slowly decomposes, as shown by the intensity of the ESR spectrum (about 10% in 30 min); TLC analysis showed formation of a myriad of compounds.

**Figure 9:**
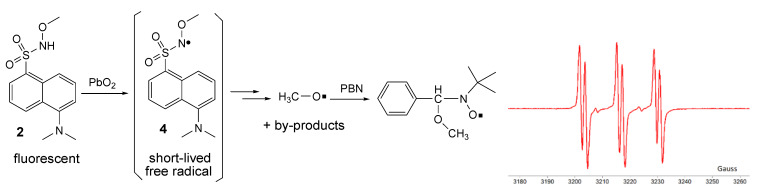
Oxidation of **2** and ESR spectrum of the spin-adduct.

By oxidation of **2** in the same conditions, no direct ESR signal could be detected; however, it is presumed that the corresponding unstable free radical **4** was formed (as for many similar compounds, the ESR spectra are showed in the literature [7,17,22]). Employing the ESR spin-trapping technique and using *t*-butyl-α-phenyl nitrone (PBN) as a spin-trap, it was possible to capture the methoxy free radical as a by-product. The recorded ESR spectrum (Figure 9) of the spin-adduct formed from PBN and the methoxy free radical consists of the well-known triplet of doublets (six lines with relatively equal intensities), with hyperfine coupling constants of *a_N_* = 13.6 G and *a_H_* = 1.9 G [25].

## 3. Materials and Methods

### 3.1. Chemicals and Apparatus

All chemicals and materials were purchased from Sigma-Aldrich (Bucharest, Romania); solvents were purchased from Chimopar (Bucharest, Romania) and used as received. TLC or column chromatography was performed using silica gel or alumina, as described in detail below. Melting points were measured using a Kruss melting point meter apparatus, using an open glass capillary.

IR spectra were recorded on a Jasco FTIR 4700 spectrophotometer (Jasco Corporation, Tokyo, Japan) using a KBr disk.

NMR spectra were recorded on Bruker Fourier 300 MHz or 500 MHz instruments (Rheinstetten, Germany), at room temperature, using deuterated chloroform or deuterated DMSO as solvents; the solvent signals were used for calibration, as internal standard.

UV-Vis spectra were recorded in dichloromethane (DCM) or ethanol at ambient temperature on dual-beam UVD-3500 or Jasco V-630 spectrometers (Labomed, LA, USA). 

High-resolution mass spectra were recorded using a Thermo Scientific LTQ XL Orbitrap spectrometer (Santa Clara, CA, USA), in positive ion mode, using the APCI technique.

X-ray diffraction measurements were performed on a STOE IPDS II diffractometer (Darmstadt, Germany), operating with a Mo-*K_α_* (λ = 0.71073 Å) X-ray tube with a graphite monochromator. The structure was solved by direct methods (using SHELXS-2013 crystallographic software, Darmstadt, Germany) and refined by full-matrix least-squares techniques based on *F^2^*. The non-H atoms were refined with anisotropic displacement parameters and hydrogen atoms were introduced at calculated positions (riding model), included in structure factor calculations but not refined. The total number of measured reflections was 16,256. Calculations were performed using the SHELXL-2018 (Darmstadt, Germany) crystallographic software package. A summary of the crystallographic data and the structure refinement for crystal **2** are given in Table 2. CCDC reference number: 1992006.

Excitation and emission spectra for compounds **1** and **2** were performed on a Thermo Scientific Varioskan Flash spectral scanning multimode reader (Santa Clara, CA, USA), in suitable plates using 5 nm excitation and emission slits for all measurements. Stock solutions of the compound were prepared in ethanol (10^−2^ M).

ESR spectra were recorded on a Jeol Jes FA 100 apparatus (Tokio, Japan), operating in the X-band. The sample was dissolved in toluene or DCM, stirred with lead dioxide, and deoxygenated by bubbling argon, and the spectrum was then recorded using classical glass NMR tubes (for toluene) and capillary tubes (for DCM). The typical settings were as follows: Frequency 8.99 GHz, center field 3220 G, sweep width 100–200 G, sweep time 60–120 s, time constant 30 ms, gain 50–500, modulation frequency 100 kHz, modulation width 1 G. For the spin-trapping experiment, few mg of **2** and PBN were mixed in toluene in the presence of lead dioxide; the supernatant was transferred to an NMR tube, deoxygenated by bubbling argon, and then recording the ESR spectrum.

### 3.2. Synthesis

*5-(Dimethylamino)-N’,N’-diphenyl-naphtalene-1-sulfonohydrazide***1.** To a solution of *N,N*-diphenylhydrazine hydrochloride (1.2 mmol, 0.265 g) in acetonitrile (40 mL), dansyl chloride (1 mmol, 0.270 g) and sodium hydrogen carbonate (5 mmol, 0.420 g) were added and the mixture was stirred at room temperature for 2 days. The mixture was filtered off and the filtrate washed with a small amount of methanol. The resulting solution was acidulated with hydrochloric acid (1 M) and extracted with DCM (3 × 100 mL), dried over anhydrous sodium sulphate, and the solvent removed under vacuum. Chromatography was applied to the residue on a silica gel column using DCM/hexane as eluent. Yields ~ 200 mg (48%). C_24_H_23_N_3_O_2_S m.w. 417. R_f_ 0.26 (silica gel, DCM) m. p. 165 °C.

IR (KBr, cm^−1^): 3236, 3061, 3038, 2984, 2943, 2866, 2831, 2876, 1590, 1495, 1455, 1405, 1331, 1270, 1162, 1147, 1075, 945, 788, 749, 694, 629, 574, 504.

^1^H-NMR (500 MHz, CDCl_3_, δ ppm, *J* Hz): 8.35 (d, *5* Hz, 1H, C*H* naphtyl); 8.25–8.22 (m, 2H, C*H* naphtyl); 7.73 (s, 1H, N*H*); 7.41 (t, *10* Hz, 1H, C*H* naphtyl); 7.33 (t, *10* Hz, 1H, C*H* naphtyl); 7.04 (d, *5* Hz, 1H, C*H* naphtyl); 6.91 (t, *7.5* Hz, 4H, C*H* phenyl); 6.91 (t, *7.5* Hz, 4H, C*H* phenyl); 6.81–6.76 (m, 6H, C*H* phenyl). ^13^C-NMR (125 MHz, CDCl_3_, δ ppm): 145.89; 133.13; 131.32; 131.22; 130.63; 129.47; 129.09; 128.23; 123.23; 122.75; 120.21; 118.63; 116.49; 114.74; 53.36; 45.14.

HR-MS (ESI+) C_24_H_23_N_3_O_2_S MW 417.52 Calculated exact mass (+H): 418.1584; found 418.1603.

*5-(Dimethylamino)-N-methoxy-naphtalene-1-sulfonamide***2.** To a solution of methoxyamine hydrochloride (3 mmol, 0.250 g) in acetonitrile (40 mL), dansyl chloride (1 mmol, 0.270 g) and sodium hydrogen carbonate (5 mmol, 0.420 g) were added and the mixture was stirred at room temperature for 1 day. The mixture was filtered off and the filtrate washed with a small amount of methanol. The resulting solution was acidulated with hydrochloric acid (1 M), extracted with DCM (3 × 100 mL), and dried over anhydrous sodium sulphate, and the solvent was removed under vacuum. The residue does not need further purification. Yields ~ 250 mg (89%). C_13_H_16_N_2_O_3_S m.w. 280. R_f_ 0.33 (silica gel, DCM). m. p. 130 °C.

IR (KBr, cm^−1^): 3103, 3008, 2991, 2956, 2897, 2841, 2765, 1589, 1568, 1463, 1411, 1343, 1293, 1166, 1149, 1037, 916, 784, 725, 621, 666, 538, 479, 415.

^1^H-NMR (500 MHz, CDCl_3_, δ ppm, *J* Hz): 8.67 (d, *10* Hz, 1H, C*H* naphtyl); 8.36 (d, *10* Hz, 1H, C*H* naphtyl); 8.30 (d, *10* Hz, 1H, C*H* naphtyl); 7.60 (t, *10* Hz, 2H, C*H* naphtyl); 7.38 (s, 1H, N*H*); 7.23 (d, *10* Hz, 1H, C*H* naphtyl); 3.69 (s, 3H, C*H*_3_O); 2.93 (s, 6H, C*H*_3_N). ^1^H-NMR (500 MHz, DMSO-d_6_, δ ppm, *J* Hz): 10.77 (s, 1H, N*H*); 8.54 (d, *8.0* Hz, 1H, C*H* naphtyl); 8.31 (d, *8.0* Hz, 1H, C*H* naphtyl); 8.20 (d, *8.0* Hz, 1H, C*H* naphtyl); 7.69 (t, *8.0* Hz, 1H, C*H* naphtyl); 7.62 (t, *8.0* Hz, 1H, C*H* naphtyl); 7.29 (d, *8.0* Hz, 1H, C*H* naphtyl); 3.60 (s, 3H, C*H*_3_O); 2.83 (s, 6H, C*H*_3_N). ^13^C-NMR (125 MHz, DMSO-d_6_, δ ppm): 151.10; 133.08; 130.60; 130.46; 129.65; 128.90; 128.13; 123.68; 119.26; 115.34; 64.40; 45.09.

Suitable crystals for X-ray diffraction measurements were obtained by the slow evaporation technique of a concentrated solution of **2** in DCM.

HR-MS (ESI+) C_13_H_16_N_2_O_3_S MW 280.34 Calculated exact mass (+H): 281.0954; found 281.0962.

*5-(Dimethylamino)-N’,N’-diphenyl-naphtalene-1-sulfonohydrazyl free radical***3.** To a solution of **1** (0.01 mmol, 4 mg) in DCM (2 mL), excess lead dioxide (100 mg) was added and the mixture was stirred for a few seconds. A color change was noticed, from almost colorless to intense purple. Filtration affords the persistent free radical **3**. ESR (toluene, Gauss*) a_N1_ = a_N2_* = 9.25. UV-Vis (DCM, nm) λ_max_ = 490 and 324.

## 4. Conclusions

Two new fluorescent compounds (**1** and **2**) containing dansyl moieties were synthesized and characterized by NMR, IR, and HR-MS. For compound **2**, the X-ray structure was obtained. Compound **1** generates by oxidation of hydrazyl persistent free radical **3**, while compound **2** generates a short-lived methoxyaminyl free radical **4** that decomposes with the formation of the methoxy free radical, which can be trapped by PBN and evidenced by ESR. Oxidation of compounds **1** and **2** quench their fluorescence. Such compounds might find further applications in free radical chemistry processes, as pro-fluorescent free radicals.

## Figures and Tables

**Table 1:** Selected bond lengths (Å) for compound **2**.

S1-O1 = 1.4201(14)	C1-C2 = 1.364(2)	C3-C4 = 1.361(2)
S1-O2 = 1.4229(14)	C2-C3 = 1.395(2)	C4-C5 = 1.407(2)
S1-N1 = 1.6601(16)	C1-C10 = 1.433(2)	C5-C6 = 1.440(2)
S1-C1 = 1.7842(16)	C9-C10 = 1.415(2)	C5-C10 = 1.432(2)
N2-C6 = 1.4301(19)	C6-C7 = 1.366(2)	C7-C8 = 1.402(2)
N2-C13 = 1.462(2)	N1-O3 = 1.4192(19)	C8-C9 = 1.359(2)
N2-C12 = 1.467(2)	N1-H1N = 0.91(2)	O3-C11 = 1.407(3)

**Table 2:** Crystallographic data and details of data collection and structure refinement parameters for compound **2.**

Compound	2
Chemical formula	C_13_H_16_N_2_O_3_S
*M* (g mol^−1^)	280.34
Temperature, (K)	293(2)
Wavelength, (Å)	0.71073
Crystal system	*Monoclinic*
Space group	*P2*_1_/*n*
*a*(Å)	12.6867(8)
*b*(Å)	7.6790(7)
*c*(Å)	14.6837(10)
*α*(°)	90
*β*(°)	109.485(5)
*γ*(°)	90
*V*(Å^3^)	1348.58(18)
Z	4
*D*_c_ (g cm^–3^)	1.381
μ (mm^–1^)	0.246
F(000)	592
Goodness-of-fit on *F*^2^	1.031
Final *R1*, *wR*_2_ [*I* > 2σ(*I*)]	0.0354, 0.0966
*R1*, *wR*_2_(all data)	0.0480, 0.1035
Largest diff. peak and hole (eÅ^–3^)	0.352, −0.277
